# Splink: Latest developments and applications

**DOI:** 10.23889/ijpds.v8i2.2245

**Published:** 2023-09-14

**Authors:** Sam Lindsay, Ross Kennedy, Tom Hepworth, Andy Bond

**Affiliations:** 1 Ministry of Justice, London, United Kingdom

Funded by ADR UK, a new data linking team at the Ministry of Justice set out to link administrative datasets across the justice space, for internal use and sharing with external researchers. To achieve this aim we sought a linkage implementation that was probabilistic, flexible, scalable and ideally open source.

Taking into account the tools available at the MoJ, existing open-source software (and paid alternatives) failed to meet our desired criteria. It was decided to develop a software package that builds on FastLink’s implementation in R of an Expectation-Maximisation algorithm to estimate a Fellegi-Sunter linkage model, adding a range of technical improvements, increased functionality and customisation options. Distributed computing offered by Spark could facilitate comparable linkage jobs that run on much larger datasets and much faster. Working with government data, accountability and transparency are vital, so the data and models are made accessible by a range of intuitive visualizations.

The Splink python package has been downloaded over 6 million times. This initially used Spark to deliver its superior performance, but Splink v3 caters for various SQL backends and more potential users. As we have made Splink more intuitive, more accessible, and more extensively documented we continue to receive feedback and contributions from around the world, driving further continuous development.

Through technical innovation and user-focused development, Splink has improved access to cutting-edge data linkage, and created groundbreaking research opportunities at MoJ and beyond. The team is grateful to ONS and other collaborators for testing and adopting these tools, and we will present some of the latest developments as well as examples of how Splink has been used worldwide.

